# Cognitive heterogeneity in mild cognitive impairment due to Alzheimer’s disease pathology

**DOI:** 10.4103/NRR.NRR-D-25-00308

**Published:** 2025-10-30

**Authors:** Siyun Chen, David P. Salmon, Howard H. Feldman, Karen Messer, Mark W. Bondi, Dongsheng Xu, Yuqi Qiu, Diane M. Jacobs, The Alzheimer’s Disease Neuroimaging Initiative

**Affiliations:** 1 Department of Rehabilitation Medicine, Shanghai Jiao Tong University Affiliated Sixth People’s Hospital, Shanghai, China; 2 Department of Neurosciences, University of California San Diego, La Jolla, CA, USA; 3 UC San Diego Shiley-Marcos Alzheimer’s Disease Research Center, University of California San Diego, La Jolla, CA, USA; 4 Alzheimer’s Disease Cooperative Study, Department of Neurosciences, University of California San Diego, La Jolla, CA, USA; 5 Division of Biostatistics and Bioinformatics, Herbert Wertheim School of Public Health, University of California San Diego, La Jolla, CA, USA; 6 Department of Psychiatry, University of California San Diego, La Jolla, CA, USA; 7 College of Rehabilitation Science, Shanghai University of Traditional Chinese Medicine, Shanghai, China; 8 KLATASDS-MOE, School of Statistics, East China Normal University, Shanghai, China

**Keywords:** Alzheimer’s disease, biomarkers, cluster analysis, cognitive heterogeneity, cognitive subtypes, dementia conversion, mild cognitive impairment, neurodegeneration, neuroimaging, neuropsychology

## Abstract

Traditional clinical subtype classifications (such as amnestic and non-amnestic mild cognitive impairment) rely on subjective interpretations of overlapping patterns of performance on cognitive tests, which may lead to unreliable categorization. A more precise and objective classification of mild cognitive impairment subtypes can be achieved through data-driven clustering techniques. However, because previous studies have not restricted their cohorts to patients who have mild cognitive impairment with the pathology of Alzheimer’s disease, the nature of cognitive variability and its impact on disease progression in a strictly defined biomarker-positive preclinical Alzheimer’s disease cohort remains unknown. We examined cognitive heterogeneity among participants with mild cognitive impairment due to Alzheimer’s disease and evaluated its prognostic utility. Neuropsychological test data from 389 patients with mild cognitive impairment in whom the cerebrospinal fluid biomarker confirmed Alzheimer’s disease were obtained from the Alzheimer’s Disease Neuroimaging Initiative cohorts. Principal component analysis and model-based clustering were used to identify cognitive profiles, which were then validated through a 100-time bootstrap analysis. Pairwise comparisons tested for differences between the identified subgroups in participant characteristics, scores on cognitive and clinical outcomes, levels of cerebrospinal fluid biomarkers, and magnetic resonance imaging-derived brain volumes. Longitudinal analyses evaluated differences in rate of change of magnetic resonance imaging volumetric measurements and clinical outcomes over 48 months. Survival analysis assessed risk for conversion to dementia. Alpha-synuclein levels and white matter hyperintensity volumes were considered for sensitivity analysis. Two distinct cognitive profiles were identified: a “typical” group (56.04% of the sample) that demonstrated relatively poorer scores on memory testing than non-memory tests, and an “atypical” group (43.96% of the sample) with smaller differences between memory and non-memory measures, indicating a more uniform pattern of impairment across cognitive domains. While the groups had comparable levels of overall cognitive impairment and cerebrospinal fluid biomarkers of Alzheimer’s disease, the typical group displayed accelerated atrophy rates every 6 months across multiple brain regions (hippocampus: 29.02 mm^3^, standard error [SE] = 10.13, *P* = 0.005; whole brain: 1799.85 mm^3^, SE = 781.57, *P* = 0.023; entorhinal cortex: 22.26 mm^3^, SE = 11.15, *P* = 0.048; fusiform gyrus: 66.24 mm^3^, SE = 28.53, *P* = 0.021). Survival analysis revealed markedly higher dementia conversion risk (hazard ratio: 1.70, 95% confidence interval: 1.27, 2.27, *P* < 0.001) and shorter progression time in the typical group. These findings persisted after controlling for comorbid pathologies. In conclusion, this data-driven approach identified two distinct cognitive subtypes of mild cognitive impairment due to Alzheimer’s disease that differed in their rates of clinical decline and neurodegeneration. These findings could be used to improve prognostic models and inform clinical trial stratification.

## Graphical abstract

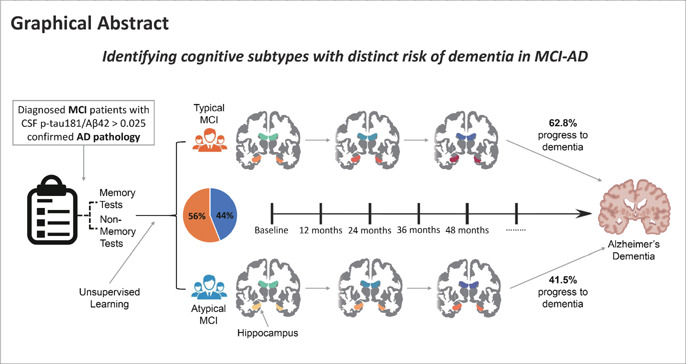

## Introduction

Mild cognitive impairment (MCI) is widely regarded as an intermediate stage of cognitive decline that falls between normal cognition and dementia (Scheltens et al., 2021; Frisoni et al., 2022). The condition is typically characterized by a relatively circumscribed memory deficit in the context of preserved mental status and independence in day-to-day functioning (Petersen et al., 2009; Petersen et al., 2013; Dubois et al., 2021; Mengel et al., 2025). Alzheimer’s disease (AD) is a common presumed cause of MCI, although it can have other neuropathological (e.g., dementia with Lewy bodies, limbic-predominant age-related TDP-43 encephalopathy [LATE], primary age related tauopathy [PART]), medical (e.g., cerebrovascular disease, infections, sleep disorders, thyroid disorders), or psychiatric (e.g., depression) causes. Numerous clinical trials have attempted to alleviate MCI and slow progression to dementia, with limited success (Huang et al., 2020, 2023; Cummings et al., 2021). Factors contributing to past failed trials may include variability in the underlying cause of MCI across trial participants or difficulty evaluating treatment effects due to cognitive heterogeneity involving impairment of language, executive functions, attention, or visuospatial abilities, either alone or in conjunction with impaired memory.

Current clinical trials at the MCI stage of AD (i.e., prodromal AD) often address etiological heterogeneity by employing cerebrospinal fluid (CSF) or plasma AD biomarkers to confirm the presumed underlying cause of impairment (Dubois et al., 2021; Altomare et al., 2023). While this reduces one aspect of heterogeneity in the targeted cohort, it may not reduce variability in the cognitive abilities most affected during the MCI phase of AD. The impact of cognitive variability might be reduced by clinically sub-categorizing MCI into amnestic and non-amnestic types with single or multiple cognitive domain subtypes (Winblad et al., 2004), but this level of clinical subtyping can be challenging because it relies on subjective interpretation of overlapping patterns of performance on cognitive tests, potentially leading to unreliable categorization. More sophisticated and objective subtyping of MCI can be achieved through data-driven clustering techniques that analyze natural groupings and similarities within a panel of neuropsychological test scores (Clark et al., 2013; Bondi et al., 2014; Eppig et al., 2017; Edmonds et al., 2021, 2024). When applied to cohorts of individuals with clinically-diagnosed MCI, the data-driven approach provides greater diagnostic precision than clinical subtyping, and results in stronger associations with AD biomarkers and more accurate prediction of decline (Bondi et al., 2014; Ferreira et al., 2020; Krell-Roesch et al., 2021; Chen et al., 2023).

Previous studies using data-driven approaches to subtype MCI have not restricted their cohort to those with biomarker confirmed AD, although they have shown stronger associations with biomarkers than conventional diagnostic schema (Clark et al., 2013; Bondi et al., 2014; Eppig et al., 2017; Edmonds et al., 2021, 2024; Shand et al., 2023). Thus, the nature of cognitive variability in a more circumscribed biomarker-positive prodromal AD cohort—and its possible implications for rate of cognitive decline, changes in neuroimaging, and ability to monitor treatment effects—remains unknown. In a past study that applied data-driven subtyping in a cohort with mild to moderate dementia due to AD, we identified two cognitive clusters: a “typical” AD cognitive profile with greater impairment to memory than to other cognitive abilities and an “atypical” AD cognitive profile in which impairment to attention/executive functions was greater and impairment to memory was less than in the typical pattern (Qiu et al., 2019). In a subsample with autopsy-confirmed AD, 79.6% of patients had the “typical” AD cognitive profile, and 20.4% had the “atypical” profile. Atypicality was associated with younger age, fewer apolipoprotein E (APOE) ε4 alleles (a variant known to confer genetic risk for AD), lower levels of neurofibrillary tangle pathology (i.e., Braak stage), less severe global dementia, higher depression scores, and slower cognitive decline. The typical or atypical cognitive profile persisted over four years of annual testing, suggesting that distinct cognitive subtypes may develop early and last throughout the course of the disease.

The present study used the Alzheimer’s Disease Neuroimaging Initiative (ADNI) cohort to explore cognitive heterogeneity and its relationship to clinical progression and neuroimaging changes in participants with MCI and biomarker-confirmed AD pathology. We focused exclusively on biomarker-confirmed AD to avoid previously identified “false positive” prodromal AD in the ADNI cohort (Edmonds et al., 2019; Elman et al., 2020). The principal component analysis (PCA) and model-based clustering approach used in Qiu et al. (2019) was applied to facilitate the identification of distinct cognitive subgroups among those with MCI due to AD. On the basis of our previous findings, we predicted that despite confirmed AD, identifiable cognitive subtypes within MCI would differ in clinical trajectories and patterns of neurodegeneration. Identification of prodromal AD cognitive subtypes may have implications for the development of more targeted and effective methods of diagnosis, monitoring treatment effects, and clinical outcomes at this pivotal stage of AD.

## Methods

### Alzheimer’s Disease Neuroimaging Initiative database

This study used data from the ADNI database. Launched in 2003, ADNI’s primary objective is to monitor the disease course of AD via a comprehensive array of indicators that includes clinical evaluation, detailed neuropsychological testing, CSF biomarkers, and advanced neuroimaging techniques. Information regarding recruitment strategies employed for the ADNI cohort can be found at www.loni.usc.edu/ADNI, and specific eligibility criteria for participation are at https://adni.loni.usc.edu/wpcontent/uploads/2024/02/ADNI_General_Procedures_Manual_29Feb2024.pdf and https://adni.loni.usc.edu/wpcontent/uploads/2024/02/ADNI2_Procedures_Manual_28Feb2024.pdf.

### Participants

Our analysis cohort comprised 389 individuals who met all inclusion criteria and none of the exclusion criteria. The inclusion criteria for the current study were: (1) Diagnosed with MCI from 3 waves of ADNI (i.e., ADNI1, ADNI2, and ADNI3). A diagnosis of MCI in the ADNI was based on meeting all of the following criteria: (a) subjective memory concern reported by the participant, study partner, or clinician; (b) abnormal memory function documented by scoring 1.5 SD below an education-adjusted cutoff-score on delayed free recall of Story A from the WMS-R Logical Memory II subtest (Reynolds and Powel, 1988); (c) Mini-Mental State Examination (MMSE) (Folstein et al., 1975) score between 24–30; (d) global Clinical Dementia Rating (CDR) (Morris, 1993) score of 0.5, with a CDR Memory Box score of at least 0.5; (e) general cognition and functional performance sufficiently preserved such that a diagnosis of dementia could not be made; (2) AD biomarker-positive CSF (p-tau181/amyloid-β [Aβ]42 > 0.025) (Hansson et al., 2018) at baseline; (3) Available baseline data for age, years of education, and APOE genotype. The exclusion criteria of the current study were: (1) No CSF biomarker data at baseline; (2) No baseline data for age, years of education, or APOE genotype; (3) Lack of results from more than 50% of the 13 neuropsychological test measures at baseline; (4) AD biomarker-negative CSF (p-tau181/Aβ_42_ ≤ 0.025).

To evaluate potential selection bias introduced by requiring CSF-biomarker data, we compared demographic and cognitive characteristics between MCI participants with and without CSF data across all ADNI waves. In the full sample, participants with CSF data were generally younger and performed better on several cognitive measures. However, when stratified by ADNI wave (ADNI1, GO, ADNI2, and ADNI3), these differences were inconsistent and typically small in magnitude. These findings suggest that although some differences exist, the risk of substantial selection bias affecting our findings is limited.

This study represents an exploratory analysis of existing observational data from the ADNI database. Therefore, the sample size was not determined by a prospective power calculation, but rather by the availability of participants meeting our inclusion/exclusion criteria. A *post hoc* power analysis was conducted to evaluate the adequacy of our sample size for detecting clinically meaningful differences between cognitive subtypes. Using the R package simr (Green and MacLeod, 2016), we determined the power for our primary longitudinal analyses via 1000 simulations. The final sample size *n* = 389 provided sufficient power for detecting group differences in brain volume changes and in dementia conversion, but was limited for detecting group differences in clinical decline.

### Ethics approval and consent to participate

The study procedures were approved by the institutional review boards of all participating centers, and written informed consent was obtained from all participants or their authorized representatives according to the *Declaration of Helsinki* (consent for research). A complete listing of all participating institutions and their institutional review boards that provided ethics approval is provided in **Additional Table 1**. The investigators within the ADNI contributed to the design and implementation of the ADNI and/or provided data but did not participate in analysis or writing of this report. A complete listing of ADNI investigators can be found online (http://adni.loni.usc.edu/wp-content/uploads/how_to_apply/ADNI_Acknowledgement_List.pdf).

**Additional Table 1:** Institutional Review Boards that provided ethics approval for the study procedures of the Alzheimer’s Disease Neuroimaging Initiative (ADNI) cohorts

Institution	Location
Oregon Health and Science University	Portland, OR, USA
University of Southern California	Los Angeles, CA, USA
University of California-San Diego	San Diego, CA, USA
University of Michigan	Ann Arbor, MI, USA
Mayo Clinic, Rochester	Rochester, MN, USA
Baylor College of Medicine	Houston, TX, USA
Columbia University Medical Center	New York, NY, USA
Washington University, St. Louis	St. Louis, MO, USA
University of Alabama at Birmingham	Birmingham, AL, USA
Mount Sinai School of Medicine	New York, NY, USA
Rush University Medical Center	Chicago, IL, USA
Wien Center	Vienna, VA, USA
Johns Hopkins University	Baltimore, MD, USA
New York University	New York, NY, USA
Duke University Medical Center	Durham, NC, USA
University of Pennsylvania	Philadelphia, PA, USA
University of Kentucky	Lexington, KY, USA
University of Pittsburgh	Pittsburgh, PA, USA
University of Rochester Medical Center	Rochester, NY, USA
University of California, Irvine	Irvine, CA, USA
University of Texas Southwestern Medical School	Dallas, TX, USA
Emory University	Atlanta, GA, USA
University of Kansas, Medical Center	Kansas City, KS, USA
University of California, Los Angeles	Los Angeles, CA, USA
Mayo Clinic, Jacksonville	Jacksonville, FL, USA
Indiana University	Indianapolis, IN, USA
Yale University School of Medicine	New Haven, CT, USA
McGill University, Montreal-Jewish General Hospital	Montreal, QC, Canada
Sunnybrook Health Sciences	Toronto, ON, Canada
U.B.C. Clinic for AD & Related Disorders	Vancouver, BC, Canada
Cognitive Neurology-St. Joseph's	Hamilton, ON, Canada
Cleveland Clinic Lou Ruvo Center for Brain Health	Las Vegas, NV, USA
Northwestern University	Chicago, IL, USA
Premiere Research Inst (Palm Beach Neurology)	West Palm Beach, FL, USA
Georgetown University Medical Center	Washington, DC, USA
Brigham and Women's Hospital	Boston, MA, USA
Stanford University	Stanford, CA, USA
Banner Sun Health Research Institute	Sun City, AZ, USA
Boston University	Boston, MA, USA
Howard University	Washington, DC, USA
Case Western Reserve University	Cleveland, OH, USA
University of California, Davis-Sacramento	Sacramento, CA, USA
Neurological Care of CNY	Liverpool, NY, USA
Parkwood Hospital	London, ON, Canada
University of Wisconsin	Madison, WI, USA
University of California, Irvine-BIC	Irvine, CA, USA
Banner Alzheimer's Institute	Phoenix, AZ, USA
Dent Neurologic Institute	Amherst, NY, USA
Ohio State University	Columbus, OH, USA
Albany Medical College	Albany, NY, USA
Hartford Hospital, Olin Neuropsychiatry Research Center	Hartford, CT, USA
Dartmouth-Hitchcock Medical Center	Lebanon, NH, USA
Wake Forest University Health Sciences	Winston-Salem, NC, USA
Rhode Island Hospital	Providence, RI, USA
Butler Hospital	Providence, RI, USA
UC San Francisco	San Francisco, CA, USA
Medical University South Carolina	Charleston, SC, USA
St. Joseph's Health Care	London, ON, Canada
Nathan Kline Institute	Orangeburg, NY, USA
University of Iowa College of Medicine	Iowa City, IA, USA
Cornell University	New York, NY, USA

University of South Florida: USF Health Byrd Alzheimer's Institute Tampa, FL, USA

### Neuropsychological test battery

Cognitive function was evaluated using a comprehensive battery of neuropsychological tests that assessed memory, attention, and executive function, visuo-constructional ability, and language (Weintraub et al., 2009; Crane et al., 2012; Hampton et al., 2023). Thirteen outcome measures from neuropsychological tests administered across all 3 ADNI waves were included in our analyses: Rey Auditory Verbal Learning Test (RAVLT) Total Immediate Recall (sum of Trials 1–5), Learning Score (Trial 5 recall minus Trial 1 recall), 30-minute Delayed Recall, and Delayed Recognition; Logical Memory Test (one story) Immediate and Delayed Recall Scores; Boston Naming Test (BNT) or Multilingual Naming Test (MINT) Total Correct; Clock Drawing Total Score; Clock Copy Total Score; Category Fluency (Animals) Total Correct; American National Adult Reading Test (ANART) Total Errors; and Time to Complete Trail Making Part A and B. Trail Making Test A and B and ANART scores were converted to make higher scores indicate better performance. Naming scores were harmonized by calculating separate z-scores for the BNT (used in ADNI 1, 2, and GO) and MINT (used in ADNI 3).

### Neuroimaging

T1-weighted magnetic resonance imaging (MRI) scans were obtained and processed using FreeSurfer software (v.5.3.0; https://surfer.nmr.mgh.harvard.edu/) to extract regional brain volume measurements across 34 gyral-based regions of interest, as defined by the Desikan-Killiany atlas (Dale et al., 1999; Fischl and Dale, 2000; Desikan et al., 2006). In this study, the analyses focused on seven AD related regions: the lateral ventricles, hippocampus, whole brain, entorhinal cortex, fusiform gyrus, middle temporal gyrus, and intracranial volume. While the FreeSurfer pipeline also generated cortical thickness measurements, our analyses focused exclusively on volumetric measures of key brain regions given their established sensitivity to neurodegeneration in early AD (Frisoni et al., 2010; Jack et al., 2010). Those regional brain volumes were analyzed in participants, with sample sizes varying between *n* = 330 and *n* = 379 across different regions. This variation is due to factors such as differences in image quality, processing success rates, and data completeness for specific brain regions.

### Cerebrospinal fluid biomarkers

The analysis cohort was restricted to participants with baseline CSF biomarker data. CSF samples were collected and analyzed according to the ADNI protocol. Biomarkers assessed included Aβ_1–42_ concentration, phosphorylated tau at threonine 181 (p-tau181), and total tau. CSF biomarkers were measured using fully automated Elecsys immunoassays on a cobas e 601 analyzer (Roche Diagnostics International Ltd, Rotkreuz, Switzerland) at the Department of Pathology & Laboratory Medicine and Center for Neurodegenerative Disease Research at the University of Pennsylvania. AD biomarker positivity was defined as a p-tau181:Aβ_42_ ratio greater than 0.025 (the cutoff described in Hansson et al., 2018).

### Statistical analysis

We derived residuals from univariate linear regressions to remove the influence of age, gender, education, and APOE genotype from each of the 13 neuropsychological outcome measures and then applied PCA to the residuals. We used the standardized residuals of cognitive test scores to reduce dimensionality and identify underlying cognitive domains. The scree plot was used to identify the “elbow” point indicating the optimal number of components to retain. The final decision to retain the components was based on their clear interpretability, which is crucial for meaningful subsequent analyses. The selected principal components extracted from the PCA were used to cluster participants into subgroups through a Gaussian model-based clustering approach with ellipsoidal, varying volume, shape, and orientation assumptions for maximum flexibility in cluster shape and size. The Expectation-Maximization (EM) algorithm was used in the clustering for parameter estimation. The optimal number of clusters was determined by comparing models with different cluster counts using the Bayesian Information Criterion and selecting the model with the lowest Bayesian Information Criterion value. PCA and clustering were performed by the “stats” and “mclust” R packages, respectively.

Following unsupervised clustering, the resulting cognitive subgroups were characterized based on their principal component (PC) scores (memory versus non-memory performance balance) and labeled post hoc according to their cognitive patterns. Clusters with memory-predominant impairment patterns were designated as “typical” MCI, while those with more uniform impairment across domains were designated as “atypical” MCI.

We employed a bootstrap resampling approach to validate the stability and reproducibility of the PCA and clustering results. Specifically, 100 bootstrap iterations were performed. In each iteration, a bootstrap sample was generated by sampling participants with replacement from the original dataset. The PCA and clustering procedures were reapplied to each resampled dataset. The loadings of the first few principal components across these bootstrap samples were visualized by forest plots in which each cognitive measure’s contribution to the components is represented by points (mean loadings) and lines (95% confidence intervals [CIs]). Agreement between the clustering solutions obtained from the bootstrap samples and the original clustering result was calculated as a co-occurrence matrix and plotted as a heatmap where each cell represents the proportion of pairs of individuals clustered together across the bootstrap iterations.

To enable comparisons across disparate scales, we calculated z-scores for the MCI sample on each of the 13 neuropsychological test measures and the various MRI-derived brain volumes. Z-scores were derived by comparing each individual’s measurement to the mean value based on a normative reference group of cognitively normal participants from the ADNI cohort, using their baseline data. Specifically, for each measurement, a linear regression model was fitted with the NTB test measurement or MRI brain volume as the outcome and age as the predictor. The regression model provided an age-adjusted expected value for each participant. The residuals from this model were then standardized to obtain Z-scores, representing the number of standard deviations each participant’s measurement deviated from the expected norm (Bethlehem et al., 2022).

Two-sample *t*-tests for continuous variables, and chi-square tests for categorical variables, were used to compare baseline demographic characteristics, MRI measurements, and scores on clinical and cognitive outcomes of empirically derived cognitive subgroups. Associations between baseline CSF biomarker levels and subgroup classification were investigated using linear regression controlling for age, gender, years of education, APOE genotype (ε4 positive/negative), and baseline MMSE score. No correction for multiple comparisons was performed because the goal of these comparisons was to identify factors underlying group membership.

Baseline MRI regional brain volumes were visualized for the cognitive subgroups and a separate group of cognitively normal individuals from the ADNI dataset. Regional volume z-scores were calculated for each participant by subtracting the mean volume of the cognitively normal group and dividing by the cognitively normal group’s standard deviation. Different views of the brain were generated by the “ggseg” package in R.

Longitudinal analyses were then conducted to evaluate differences between the identified cognitive subgroups in the rate of decline over 48 months on clinical outcomes such as the Alzheimer’s Disease Assessment Scale-Cognition (ADAS-Cog13) (Rosen et al., 1984), Clinical Dementia Rating-Sum of Boxes (CDR-SB) (Morris, 1993), MMSE, and MRI regional brain volumes. High rates of missing evaluations precluded analysis of longer follow-up periods. Respons-profile models incorporating a categorical time variable were applied to capture non-linear trajectories, and the unstructured covariance matrix was assumed. Linear mixed-effects models were applied for outcomes exhibiting linear trajectories. These models were adjusted for age, gender, years of education, APOE genotype, baseline levels of the respective outcome, and the subgroup-by-time interaction. Regression models for MRI volume change also controlled for baseline intracranial volume to correct for head size. Response-profile modeling was performed by the R package “gls” and mixed-effects modeling was performed by the “lme4” package. A power analysis was performed using the R package “simr”, which incorporated a 1000-times simulation of the fitted longitudinal model (Green and MacLeod, 2016).

Survival analyses were used to test for differences between cognitive subgroups in the rate of progression from MCI to dementia. The Kaplan-Meier method and log-rank tests supported comparison of unadjusted survival curves between subgroups. A Cox proportional hazards model (R package: “survival”), adjusting for age, gender, years of education, APOE genotype, and baseline cognitive performance, was used to determine if the risk of progression to AD differed between cognitive subgroups.

To explore the potential influence of comorbid pathologies on our results, we compared baseline CSF alpha-synuclein levels, a marker of potential Lewy body pathology; white matter hyperintensity (WMH) volumes, a marker of cerebrovascular disease; and depressive symptoms quantified by the Geriatric Depression Scale (GDS) (Yesavage et al., 1982) between the cognitive subgroups. Sensitivity analyses were conducted where alpha-synuclein level, WMH volume, or GDS total score were separately used as covariates in all the regression models.

Participants were excluded from our analyses if results from more than 50% of the 13 neuropsychological test measures were missing at baseline. Otherwise, multiple imputation was used for missing neuropsychological test scores. Subsequent analyses were conducted using a complete case analysis approach for all variables relevant to each specific analysis.

All analyses were performed with R version 4.4.0 (https://www.r-project.org/).

## Results

### Participant characteristics, baseline cerebrospinal fluid measurements, and brain volumes

**Figure 1:**
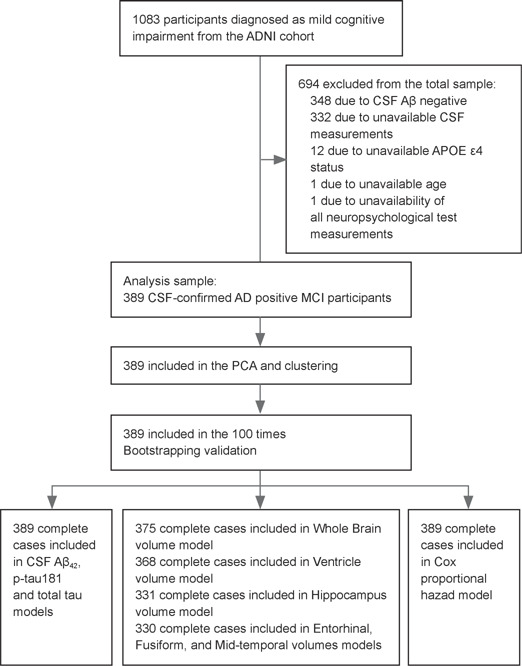
Flow diagram of participants included in the main analyses. This represents a cross-sectional analysis with longitudinal follow-up of existing Alzheimer’s Disease Neuroimaging Initiative (ADNI) data. AD: Alzheimer’s disease; Aβ: amyloid-β; CSF: cerebrospinal fluid; MCI: mild cognitive impairment; p-tau181: phosphorylated tau at threonine 181.

The mean age, years of education, MMSE, CDR-SB, and ADAS-Cog13 of the 389 ADNI participants included in this study are shown in **Figure 1**. There were 233 (59.90%) men and 272 (69.92%) APOE ε4 carriers (**Table 1**). Twelve of the 389 included participants were missing one of the 13 cognitive test scores at baseline; none was missing more than one baseline cognitive test score. Mean levels of CSF biomarkers and MRI-derived brain volumes for various ROIs at baseline are summarized in **Table 1**.

**Table 1:** Participant characteristics for the overall sample and MCI cognitive subtypes

	Overall	Typical MCI	Atypical MCI	*t* or χ^2^ value	*P* value
*n*	389	218	171		
Age (yr)	73.46±7.08	73.41±6.78	73.54±7.46	0.18	0.857
Sex (male)	233 (59.90)	135 (61.93)	98 (57.31)	0.67	0.413
Education (yr)	15.94±2.81	15.87±2.85	16.04±2.77	0.58	0.559
APOE ε4 carrier	272 (69.92)	152 (69.72)	120 (70.18)	0	1
CDR-SB	1.64±0.95	1.63±0.97	1.65±0.93	0.22	0.826
ADAS-Cog13*	18.57±6.68	20.022±5.50	16.73±7.55	4.96	< 0.001
MMSE	27.32±1.96	27.21±1.93	27.46±1.98	1.25	0.212
**Neuropsychological test scores**					
RAVLT Immediate Recall (Trials 1–5)*	31.38±9.00	28.59±6.70	34.92±10.24	7.34	< 0.001
RAVLT Learning (Trial 5 - 1)*	3.51±2.43	2.50±1.79	4.80±2.54	10.45	< 0.001
RAVLT Forgetting (Trial 5 - Delayed Recall)*	4.96±2.26	5.39±2.01	4.43±2.45	4.24	< 0.001
RAVLT 30-Min. Delayed Recall*	2.74±3.04	1.30±1.65	4.57±3.41	12.42	< 0.001
RAVLT Delayed Recognition*	9.71±3.48	8.38±3.13	11.40±3.17	9.39	< 0.001
Logical Memory Immediate Recall*	7.74±3.48	7.05±3.14	8.62±3.70	4.52	< 0.001
Logical Memory Delayed Recall*	4.99±3.51	3.76±3.11	6.56±3.38	8.47	< 0.001
Boston Naming Total Correct*	25.89±3.87	26.64±2.74	24.92±4.82	4.17	< 0.001
Multilingual Naming Test	28.63±5.77	28.79±6.41	28.45±5.12	0.2	0.846
Category Fluency (Animals)*	16.67±4.91	17.22±4.61	15.98±5.19	2.48	0.014
Clock Drawing*	4.20±0.98	4.35±0.85	4.02±1.09	3.37	0.001
Clock Copy*	4.60±0.72	4.78±0.47	4.37±0.90	5.78	< 0.001
ANART (errors)*	13.30±9.44	12.39±9.12	14.45±9.74	2.14	0.033
Trail Making A (Time in seconds)*	44.57±22.33	39.05±13.44	51.61±28.62	5.73	< 0.001
Trail Making B (Time in seconds)*	126.12±69.35	106.36±47.92	150.99±83.00	6.6	< 0.001
**CSF biomarker level†**					
Tau (pg/mL)	349.32±130.51	349.95±128.67	348.52±133.20	0.11	0.915
p-tau 181 (pg/mL)	35.57±14.97	35.75±14.83	35.34±15.19	0.27	0.786
Aβ_42_ (pg/mL)	653.77±210.52	652.14±206.83	655.85±215.72	0.17	0.863
Aβ_40_ (pg/mL)	18325.08±5274.13	18114.87±5059.24	18610.36±5586.25	0.53	0.596
Aβ_42/40_ ratio	0.04±0.01	0.04±0.01	0.04±0.01	0.95	0.344
Neurofilament light chain (pg/mL)	1485.64±712.97	1497.42±764.19	1463.12±610.70	0.26	0.795
Neurogranin (pg/mL)	580.02±367.72	605.99±405.49	530.38±279.35	1.12	0.265
**MRI volumes‡**					
Lateral ventricles (mm3)	41118.23±22163.24	41016.65±22570.49	41250.29±21692.18	0.1	0.92
Hippocampus (mm3)*	6594.12±1028.25	6398.38±996.37	6854.64±1015.47	4.09	< 0.001
Whole brain (mm3)	1032861.65±109090.13	1027478.01±99066.10	1039788.17±120731.47	1.08	0.279
Entorhinal (mm3)*	3469.86±742.60	3338.15±721.24	3646.41±736.59	3.81	< 0.001
Fusiform (mm3)	17504.19±2751.16	17490.64±2649.82	17522.36±2890.91	0.1	0.918
MidTemp (mm3)	19402.22±2959.57	19311.81±2684.06	19523.39±3299.06	0.64	0.521
Intracranial volume (mm3)	1538074.83±167999.49	1534133.35±157837.54	1543132.28±180564.14	0.52	0.606

Participant characteristics and baseline neuropsychological test scores, Alzheimer’s disease biomarker levels in CSF, and MRI-derived brain volumes for the overall sample and MCI cognitive subtypes. Means, standard deviations, and tests of differences between MCI cognitive subgroups (with P-values) are shown. Statistics are expressed as mean ± SD and n (%) for continuous variables and categorical variables, respectively. Two-sample t-tests and chi-square test were used for continuous variables and categorical variables, respectively. *Indicates significant group difference (P < 0.05). †132 participants (76 typical vs. 56 atypical) with CSF Aβ_40_ and Aβ_42/40_ ratio, 131 (86 vs. 45) with neurofilament light chain and neurogranin. All 389 participants had total tau, phosphorylated tau 181, and Aβ_42_. ‡Participants (typical vs. atypical) with MRI volumetric measures were 368 (208 vs. 160) for lateral ventricles, 331 (189 vs. 142) for hippocampus, 375 (211 vs. 164) for whole brain, 330 (189 vs. 141) for entorhinal, 330 (189 vs. 141) for fusiform, 330 (189 vs. 141) for mid-temporal, and 379 (213 vs. 166) for intracranial volume. ADAS-Cog13: Alzheimer’s Disease Assessment Scale-Cognition; ANART: American National Adult Reading Test; APOE: apolipoprotein E; Aβ: amyloid-β; CDR-SB: Clinical Dementia Rating-Sum of Boxes; CSF: cerebrospinal fluid; MCI: mild cognitive impairment; MMSE: Mini-Mental State Examination; MRI: magnetic resonance imaging; RAVLT: Rey Auditory Verbal Learning Test.

### Principal component analysis of neuropsychological test scores

Two distinct principal components with mean zero were identified, accounting for 46.10% of the total variance in the analysis sample (**Table 2**). A positive PC score signifies that the variables are positively correlated and increase or decrease at the same time, whereas a negative score suggests that the variables are negatively associated, so that one decreases while the other increases. The first principal component (PC1) represents “cognitive severity” because it showed consistently positive loadings across all cognitive measures (**Table 2**), capturing shared variance that reflects overall level of cognitive functioning. Higher PC1 scores indicate better overall performance across all tests while lower scores indicate poorer overall performance, independent of the specific pattern of strengths and weaknesses reflected in PC2. Conversely, the second principal component (PC2) showed positive loadings for tests assessing episodic memory, but negative loadings for tests unrelated to memory. Hence, a higher PC2 score indicates comparatively superior memory-related cognitive abilities and inferior non-memory-related cognitive abilities. Thus, PC2 functions as a nuanced metric that differentiates cognitive profiles within the spectrum of MCI, independent of overall level of cognitive impairment.

**Table 2:** Results of the principal component analysis for cognitive test scores of participants with mild cognitive impairment due to Alzheimer’s disease

Overall variance explained	46.10%†	
	PC1	PC2
RAVLT Immediate Recall (Sum of Trials 1–5)	0.4	0.14
RAVLT Learning (Trial 5 minus Trial 1)	0.32	0.26
RAVLT 30-min Delayed Recall	0.34	0.35
RAVLT Delayed Recognition	0.29	0.27
Logical Memory Immediate Recall	0.32	0.10
Logical Memory Delayed Recall	0.33	0.23
Naming Test*	0.23	–0.3
Clock Drawing	0.19	–0.26
Clock Copy	0.14	–0.34
Category Fluency	0.28	–0.24
ANART (Total Correct)	0.19	–0.24
Trail Making A (Time to Completion)	0.19	–0.35
Trail Making B (Time to Completion)	0.25	–0.37

Principal components 1 (PC1) and 2 (PC2) are shown. †Percentage of total variance in the 13 neuropsychological variables captured by the first two principal components. *Naming test indicates the Boston Naming Test or the Multilingual Naming Test. Details about how the scores from the two tests were combined are described in the Methods section. ANART: American National Adult Reading Test; RAVLT: Rey Auditory Verbal Learning Test.

**Figure 2:**
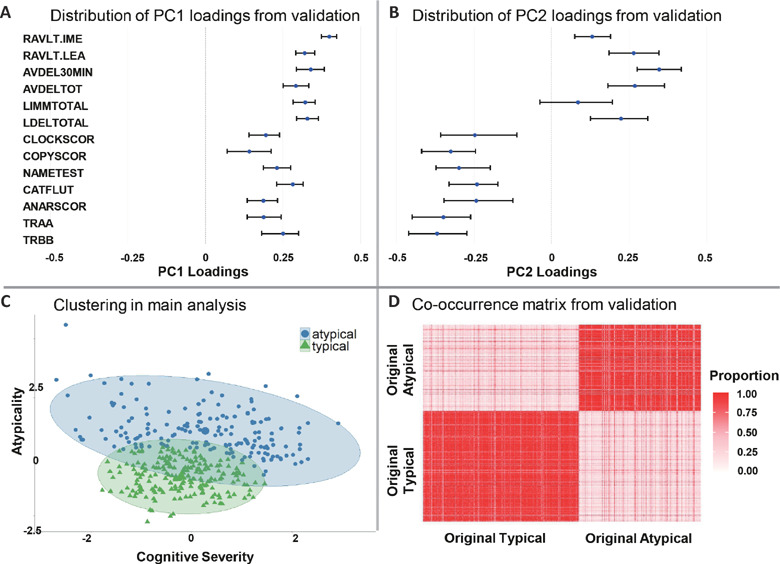
Classification of cognitive heterogeneity in patients with MCI. Principal component analysis was performed on standardized residuals of 13 neuropsychological test scores after the effects of age, sex, education, and apolipoprotein E genotype were removed. Model-based clustering using Gaussian mixture models with ellipsoidal, varying volume, shape, and orientation assumptions. Optimal cluster number determined by the Bayesian information criterion. (A, B) Forest plots of the PC1 (A) and PC2 (B) loadings for NTB measurements through 100 times Bootstrap validation, respectively. The blue dots indicate the means of the loadings, and the lines indicate the 95% confidence intervals of the loadings. (C) Clustering of typical (green) and atypical (blue) MCI participants by cognitive severity and atypicality in the main analysis sample. The y-axis labeled “atypicality” represents the PC2 score, which quantifies the relative balance between memory and non-memory cognitive performance. Higher PC2 values (upper portion) indicate a cognitive profile with relatively preserved memory function compared with that in non-memory domains, while lower PC2 values (lower portion) indicate relatively greater impairment in memory compared with that in non-memory domains. This dimension captures the key pattern differentiating typical (memory predominant) from atypical (more uniform impairment) MCI, independent of overall cognitive impairment severity, which is represented on the x-axis. (D) Heatmap of the co-occurrence matrix from 100 bootstrap iterations. Each cell represents the proportion of times (0–1) that a pair of individuals was clustered together across bootstrap samples. The red blocks along the diagonal demonstrate high within-cluster stability, where participants originally classified in the same group (typical-typical or atypical-atypical) were consistently clustered together in bootstrap iterations. The white off-diagonal areas indicate that participants who were originally classified into different groups rarely clustered together in subsequent iterations, confirming the robustness of the two-cluster solution. ANARSCOR: American National Adult Reading Test total correct; AVDEL30MIN: RAVLT 30-Minute Delayed Recall; AVDELTOT: RAVLT: Delayed Recognition; CATFLUT: Category Fluency (Animals); CLOCKSCOR: Clock Drawing; COPYSCOR: Clock Copy; LDELTOTAL: Logical Memory Delayed Recall; LIMMTOTAL: Logical Memory Immediate Recall; MCI: mild cognitive impairment; NAMETEST: Naming Test; PC1: principal component 1; PC2: principal component 2; RAVLT. IME: RAVLT Immediate Recall; RAVLT. LEA: RAVLT Learning; TRAA: Trail Making Test A, time to completion; TRBB: Trail Making Test B, time to completion.

The PCA demonstrated robust results across bootstrapped validation samples (**Figure 2A** and **B**). Factor loadings for the first two principal components remained stable throughout the validation process. In 100 bootstrap iterations, the average PC1 loadings for all NTB assessments were positive and closely aligned with the original loadings presented in **Table 2**. The 95% CIs derived from bootstrapping indicated that all 13 loadings were significantly greater than 0 (**Figure 2A**), supporting the interpretation of PC1 as a measure of overall cognitive impairment. PC2 loading patterns were similarly replicated across the bootstrap samples, with mean values approximating the original PC2 loadings. The 95% CIs for PC2 loadings revealed that all memory-related measures, except Logical Memory Immediate Recall, had loadings significantly above 0, while all non-memory measures had loadings significantly below 0 (**Figure 2B**). These findings again suggest that PC2 represents a robust, nuanced metric that distinguishes between cognitive profiles within the MCI (i.e., prodromal AD) spectrum, independent of overall level of cognitive impairment.

### Classifying clinical subgroups based on neuropsychological test scores

**Figure 3:**
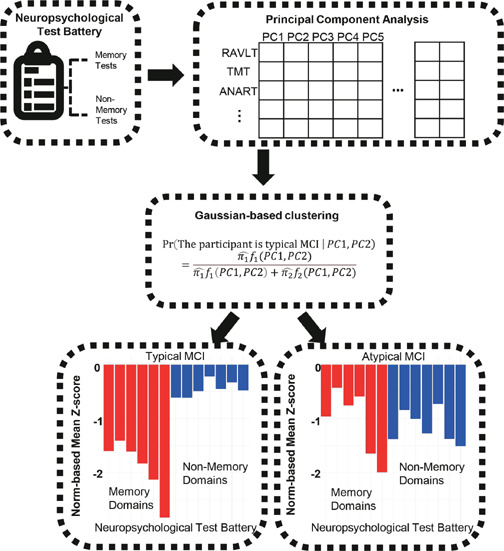
Flowchart of the classification process. Gaussian mixture model classification was performed using bivariate normal distributions. The classification probability was calculated using likelihood ratios with a threshold of 0.5. The middle box shows the decision rule of the classification. The bottom boxes display the average Z scores of each neuropsychological test. The red bars represent memory-related tests, whereas the blue bars represent non-memory tests. The likelihood that an incoming participant represents a typical case of MCI is calculated via the formula in the middle box. This probability is then evaluated against a threshold of 0.5; participants with a probability above this threshold are classified as typical MCI, whereas those below it are deemed atypical MCI. Within our analysis cohort, the estimated proportions and are 0.52 and 0.48, respectively. The density functions for the typical and atypical groups, *f*_1_ and *f*_2_, follow bivariate normal distributions with means *μ*_1_ = (–0.43, 0.43) and *μ*_2_ = (–0.97, 0.96) and covariance matrices 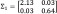 and 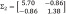. From left to right, the neuropsychological test battery includes RAVLT immediate recall, RAVLT learning, RAVLT 30-minute delayed recall, RAVLT delayed recognition, logical memory immediate recall, logical memory delayed recall, naming test, category fluency, clock drawing, clock copy, American National Adult Reading Test (ANART) (total correct), Trail Making A and Trail Making B (time to completion). MCI: Mild cognitive impairment; PC: principal component; RAVLT: Rey Auditory Verbal Learning Test.

Model-based clustering of factor scores for cognitive severity (PC1) and cognitive profile (PC2) was used to identify distinct neuropsychological groups. This analysis revealed two clusters (**Figure 2C**): Cluster 1 (218 participants, 56.04% of the sample) scored below average on the cognitive profile factor (PC2, mean = –0.96) and modestly below average on cognitive severity (PC1, mean = –0.42); Cluster 2 (171 participants, 43.96% of the sample) scored above average on the cognitive profile factor (mean 1.22) and modestly above average on cognitive severity (mean = 0.54). These distinct cognitive profiles were observed across the spectrum of severity in this MCI cohort. Cluster 1 represented a typical amnestic MCI cognitive pattern with prominent memory impairment, while Cluster 2 represented an atypical MCI cognitive pattern with a different profile, characterized by less disparity between memory and non-memory performance compared with the typical group. Bar plots of norm-based z-scores for each cognitive test measure at the bottom of **Figure 3** clearly show that the typical group displayed a non-uniform pattern of impairment with scores on memory-related measures being worse than those on other cognitive measures. In contrast, the atypical group—representing nearly half of the MCI participants—has a more uniform pattern of impairment across cognitive domains, with smaller differences between memory and non-memory measures, despite both groups having been diagnosed with amnestic MCI via ADNI criteria.

The same clustering methodology was used when performing the bootstrapped validation. The co-occurrence matrix, visualized as a heatmap (**Figure 2D**), represents the frequency with which pairs of individuals were clustered together across bootstrap iterations, with values ranging from 0 (never clustered together, shown in white) to 1 (always clustered together, shown in red). The distinct block structure indicates high within-cluster consistency and stability wherein individuals from the same original subgroup are typically clustered together across bootstrap iterations while individuals from different original subgroups are rarely clustered together in subsequent iterations. This clear delineation in the co-occurrence matrix provides robust evidence for the distinctiveness of the two identified subgroups.

### Demographic and clinical characteristics of neuropsychological subtypes

**Table 1** presents demographic characteristics, clinical and neuropsychological test scores, CSF biomarker values, and MRI volumetric measures for the typical and atypical subgroups. The subgroups did not differ in demographic characteristics (i.e., age, gender, education); genetic risk for AD (i.e., frequency of APOE ε4 alleles); overall mental status (MMSE); or day-to-day functioning (CDR-SB), suggesting that the observed cognitive heterogeneity could not be attributed to differences in participant characteristics, overall mental status, functional ability, or genetic AD susceptibility. However, the atypical subgroup performed significantly better than the typical subgroup on all neuropsychological tests of memory, and significantly worse on the non-memory tests (**Table 1**; all *P*-values < 0.05, *t*-values ranged from 2.13 to 12.40, except for MINT total score where the *P*-value was 0.846 and the *t*-value was 0.20). The atypical subgroup also performed significantly better than the typical subgroup on the ADAS-Cog13 (mean difference = 3.29, 95% CI [1.99, 4.59], *t* = 4.96, *P* < 0.001), likely owing to the heavy learning and memory load of this measure. Group comparisons of ADAS-Cog13 subtest scores confirmed that the atypical group performed significantly better than the typical group on memory-dependent subtests (Word Recall, Delayed Word Recall, Word Recognition, Orientation; all *P*-values < 0.005, all *t*-values > 2.29; **Additional Table 2**), whereas the typical group performed significantly better than the atypical group on non-memory measures (Commands, Auditory Comprehension, Word Finding Difficulty, Number Cancellation; all *P*-values < 0.020, *t*-values > 2.33; **Additional Table 2**). No significant differences were observed on the ADAS-Cog13 Constructional Praxis, Naming, Ideational Praxis, Spoken Language, or Remembering Test Instructions subtests.

**Additional Table 2:** Comparisons of ADAS-Cog13 subtest scores between the identified MCI subgroups

	Typical MCI	Atypical MCI	*t* or χ^2^ value	*P* value
N	218	171		
Word Recall	4.95±1.30	4.46±1.61	3.30	0.001
Commands	0.13±0.42	0.26±0.50	2.76	0.006
Constructional Praxis	0.52±0.56	0.60±0.60	1.25	0.214
Delayed Word Recall	7.05±2.06	5.38±2.43	7.33	<0.001
Naming Objects and Fingers	0.22±0.43	0.30±0.60	1.68	0.094
Ideational Praxis	0.11±0.36	0.12±0.35	0.23	0.822
Orientation	0.72±0.96	0.50±0.95	2.29	0.023
Word Recognition	5.20±2.65	3.39±2.64	6.72	<0.001
Remembering Test Instructions	0.09±0.39	0.10±0.40	0.19	0.848
Auditory Comprehension	0.05±0.23	0.13±0.45	2.33	0.02
Word-Finding Difficulty	0.21±0.45	0.37±0.70	2.86	0.004
Spoken Language Ability	0.08±0.34	0.11±0.39	0.73	0.466
Number Cancellation	0.72±0.88	1.02±1.07	3.04	0.003

Higher scores indicate worse performance. Statistics are expressed as mean ± SD for continuous variables. ADAS-Cog13: Alzheimer’s Disease Assessment Scale-Cognition; MCI: mild cognitive impairment.

### Cerebrospinal fluid biomarker levels of neuropsychological subtypes

There were no significant differences in baseline CSF levels for total tau, p-tau181, or Aβ_42_ between the typical and atypical subgroups, nor were there significant differences between the typical and atypical subgroups in levels of CSF Aβ_40_, the Aβ_42/40_ ratio, NfL, or neurogranin in a subset of participants for whom these additional biomarkers were available (see **Table 1** for additional detail). This lack of difference between subgroups persisted after adjusting for potential confounding factors such as age, gender, years of education, APOE genotype, and baseline MMSE score in linear regression models. These results indicate that cognitive heterogeneity can exist within a population of MCI individuals with similar biomarker profiles.

### Baseline and longitudinal regional brain volumes of neuropsychological subtypes

**Figure 4:**
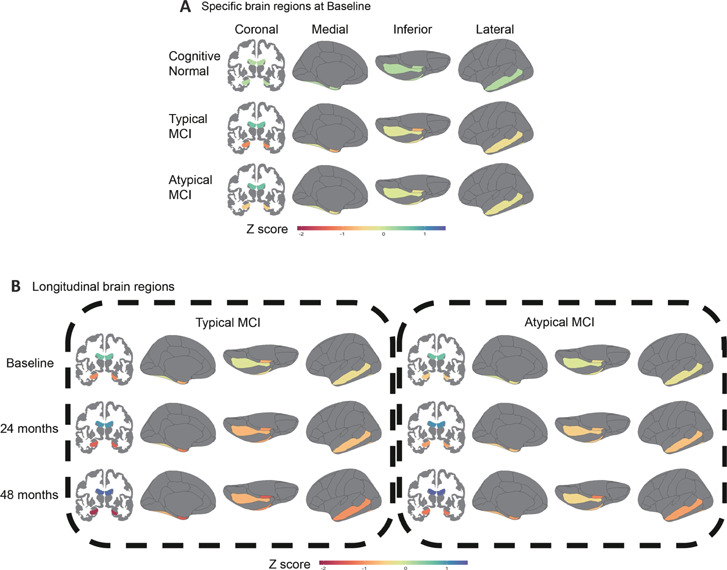
Brain regional MRI volumes in different views. Specific brain regions include the hippocampus, lateral ventricles, entorhinal cortex, fusiform gyrus, and midtemporal regions. Color indicates volume in norm-based Z scores, from relatively smaller (red) to relatively larger (blue). Z scores represent the standardized deviation of an individual’s brain regional volume from the mean volume of a normative control group, adjusted for age. Negative Z scores indicate volumes smaller than the normative mean, suggesting atrophy, whereas positive Z scores indicate larger volumes. The normative dataset was derived from cognitively normal participants within the ADNI cohort at baseline. Thus, the Z scores of the cognitively normal participants were 0 for all regions. (A) Baseline brain volumes among the cognitively normal, atypical MCI, and typical MCI groups. Two-sample *t*-tests comparing baseline volumes between cognitive subgroups. (B) Longitudinal brain volumes of the typical and atypical MCI groups at baseline, 24 months, and 48 months. Linear mixed-effects models for longitudinal analysis adjusted for age, sex, years of education, APOE genotype, baseline ROI volume, baseline intracranial volume, and subgroup-by-time interaction. ADNI: Alzheimer’s Disease Neuroimaging Initiative; MCI: mild cognitive impairment; MRI: magnetic resonance imaging; ROI: region of interest.

**Figure 4A** illustrates baseline volumetric measurements in multiple brain views for typical and atypical MCI subgroups and an age-adjusted cognitively unimpaired control group from the ADNI cohort. The MCI subgroups had lower volumes in all measured brain regions compared with the cognitively unimpaired group (**Figure 4A** and **Table 1**). Average hippocampal and entorhinal cortex volumes were significantly smaller in the typical MCI subgroup than in the atypical MCI subgroup (hippocampus: mean difference = 456.25 mm^3^, 95% CI [236.78, 675.73], *t* = 4.09, *P* < 0.001; entorhinal cortex: mean difference = 308.26 mm3, 95% CI [148.93, 467.59], *t* = 3.81, *P* < 0.001). The two MCI subgroups did not differ in baseline whole-brain, lateral ventricular, fusiform gyrus, mid-temporal area, or intracranial volume.

We examined the neurodegeneration trajectory within the typical and atypical MCI subgroups with longitudinal analyses that incorporated linear mixed-effects models that were adjusted for age, gender, years of education, APOE genotype, baseline ROI volume, baseline intracranial volume, and the subgroup-by-time interaction. **Figure 4B** illustrates model-predicted volumetric measurements in multiple brain views at baseline, 24 months, and 48 months for the typical and atypical MCI subgroups. While volume in the assessed regions declined over time in both groups, the typical MCI subgroup experienced a more rapid decrease than the atypical subgroup in the hippocampus, whole brain, entorhinal cortex, and fusiform gyrus. This accelerated decline was quantified by regression models, which revealed significantly faster decline in hippocampal volume in the typical MCI subgroup, with an additional loss of 29.02 mm^3^ every 6 months (SE = 10.13, *t* = −2.86, *P* = 0.005) in the typical MCI subgroup compared with the findings in the atypical MCI subgroup. Similar patterns of accelerated decline in volume were observed in the whole brain (additional loss: 1,799.85 mm^3^ every 6 months, SE = 781.57, *t* = −2.30, *P* = 0.023), entorhinal cortex (additional: 22.26 mm³ every 6 months, SE = 11.15, *t* = −2.00, *P* = 0.048) and fusiform gyrus (additional loss: 66.24 mm^3^ every 6 months, SE = 28.53, *t* = −2.32, *P* = 0.021). These findings suggest heightened vulnerability of brain regions associated with memory function to neurodegenerative processes in the typical MCI subgroup.

### Difference in time to progression to dementia between neuropsychological subtypes

**Figure 5:**
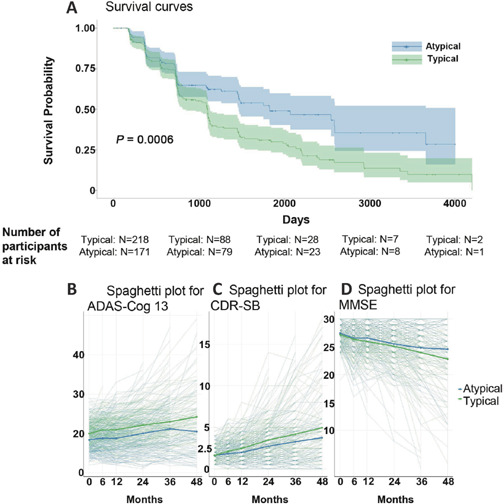
Survival curves and Spaghetti plots between neuropsychological subtypes. (A) Kaplan‒Meier survival analysis with the log-rank test for group comparisons. Survival curves for time to conversion to dementia for the typical (green) and atypical (blue) MCI groups. The unit of time (x-axis) is months. The table under the curves indicates the number of participants at risk over time between the typical and atypical groups. Spaghetti plots over 48 months for the ADAS-Cog13 total score (B), CDR-SB score (C), and MMSE total score (D) in the typical (green) and atypical (blue) MCI groups. Linear mixed effects models adjusted for age, sex, years of education, APOE genotype, baseline outcome level, and the subgroup-by-time interaction were fitted for the three outcomes. ADAS-Cog13: Alzheimer’s Disease Assessment Scale-Cognition 13-item version; APOE: Apolipoprotein E; CDR-SB: Clinical Dementia Rating-Sum of Boxes; MCI: mild cognitive impairment; MMSE: Mini-Mental State Examination.

Survival analysis revealed that the typical MCI group exhibited faster progression to dementia than the atypical group. Overall, the median follow-up duration for the 389 participants was 757 days with an interquartile range of 1080 days. Kaplan-Meier curves (**Figure 5A**) underscore this distinction with a notable divergence between the two groups’ survival curves beyond 36 months where the typical MCI subgroup’s curve steeply declines relative to the more gradual descent for the atypical MCI subgroup. A Cox proportional hazards model, adjusted for age, gender, years of education, and APOE genotype, revealed that the typical subgroup faces a substantially greater risk of advancing to AD dementia than the atypical subgroup, with a hazard ratio of 1.70 (95% CI: [1.27, 2.27], *z* = 3.57, *p* < 0.001). These results demonstrate the prognostic value of cognitive subtyping among individuals with MCI due to AD.

### Longitudinal trajectories in clinical outcomes between neuropsychological subtypes

Longitudinal change in ADAS-Cog13 total score (**Figure 5B**), CDR-SB score (**Figure 5C**), and MMSE total score (**Figure 5D**) over 48 months is displayed in **Figure 5**. Linear mixed effects models adjusting for age, gender, years of education, APOE genotype, baseline outcome level, and the subgroup-by-time interaction were fitted for the three outcomes. The ADAS-Cog13 model revealed significantly worse baseline scores for the typical MCI subgroup than for the atypical MCI subgroup (model-fitted difference = 3.82, SE = 0.63, *t* = 6.02, *P* < 0.001), but no subgroup-by-time interaction effect. There were no significant between-group differences in baseline for CDR-SB or MMSE scores (model-fitted difference = 0.14 and 0.29, SE = 0.10 and 0.20, *t* =1.39 and 1.45, *P* = 0.165 and 0.148, respectively), and no subgroup-by-time interaction effects. Although none of the models revealed statistically significant subgroup-by-time interaction terms, the CDR-SB model showed a trend toward differential rates of change with the typical MCI subgroup showing more rapid worsening than the atypical MCI subgroup (model-fitted difference = 0.09, SE = 0.05, *t* = 1.70, *P* = 0.090), and visual inspection of 48-month change in CDR-SB (**Figure 5C**) was consistent with this trend. It may be the case, however, that the study not powerful enough to detect this effect because the power analysis indicated that a sample size of 1050 would be required to detect a group difference of 0.09 in CDR-SB change with 80% power at *α* = 0.05 (**Additional Figure 1**).

### Sensitivity analyses

To explore the potential influence of comorbid pathologies, we examined baseline CSF alpha-synuclein level, WMH volume, and depressive symptomatology across the derived MCI subgroups. CSF alpha-synuclein was available for 129 of the 389 participants (typical; *n* = 85; atypical: *n* = 44) and showed no significant difference between typical and atypical MCI subgroups (mean difference = 0.13 ng/mL, 95% CI [−0.11, 0.36], *t* = 1.07, *P* = 0.289). WMH volumes were available for 251 participants (typical: *n* = 128; atypical: *n* = 123) and were significantly higher in the atypical MCI subgroup than in the typical MCI subgroup (mean difference = 2.71, 95% CI [0.35, 5.06], *t* = 2.27, *P* = 0.025). Notably, controlling for WMH volume as a covariate in all regression models did not alter the original findings from our main sensitivity analyses (**Additional Table 3**). GDS total score was available for all 389 participants and did not significantly differ between the two groups (mean difference = −0.23, 95% CI [−0.51, 0.05], *t* = −1.61, *P* = 0.108). Sensitivity analyses of the GDS total score showed similar results to the main analyses.

**Additional Table 3:** Comparisons between primary analysis and WMH-adjusted sensitivity analysis

Analysis type	Outcome measure	Primary analysis results	WMH-adjusted results
CSF biomarker levels (linear regression models)	Tau (pg/mL) (typical vs. atypical)	3.17 [-24.27,30.71], *t*=0.23, *P*=0.821	-6.87 [-43.18, 29.44], *t*=-0.37, *P*=0.709
	P-Tau 181 (pg/mL) (typical vs. atypical)	0.95 [-2.17, 4.07], *t*=0.60, *P*=0.551	-0.86 [-4.94,3.21], *t*=-0.42, *P*=0.676
	Aβ42/40 ratio (typical vs. atypical)	0[0, 0], *t*=0.86, *P*=0.390	0[0, 0], *t*=1.11, *P*=0.268
MRI brain volumes longitudinal change (mixed-effects models)	Lateral ventricular expansion (per 6 months)	-0.14 [-313.19, 312.90], *t*=0, *P*=0.999	51.88 [-315.12,418.88], *t*=0.28, *P*=0.782
	Hippocampal volume loss (per 6 months)	-29.02 [-48.87, -9.16], *t*=-2.86, *P*=0.005	-28.75 [-55.71,-1.79], *t*=-2.09, *P*=0.039
	Whole brain volume loss (per 6 months)	-1799.85 [-3331.7, -267.99], *t*=-2.3, *P*=0.023	-1737.48 [-3382.44, -92.52], *t*=-2.07, *P*=0.04
	Entorhinal cortex loss (per 6 months)	-22.26 [-44.12, -0.40], *t*=-2.00, *P*=0.048	-32.77 [-58.49, -7.05], *t*=-2.50, *P*=0.015
	Fusiform gyrus loss (per 6 months)	-66.24 [-122.15,-10.33], *t*=-2.32, *P*=0.021	-46.39 [-118.00, 25.23], *t*=-1.27, *P*=0.207
	Middle temporal gyrus loss (per 6 months)	-61.9 [-140.72, 16.91], *t*=-1.54, *P*=0.126	-40.08 [-118.89,38.74], *t*=-1.00, *P*=0.321
Dementia conversion (Cox proportional hazard model)	Hazard ratio (typical vs. atypical)	1.70 [1.27, 2.27], *z*=3.57, *P*<0.001	1.63 [1.11, 2.38], *z*=2.50, *P*=0.013

In both the primary and sensitivity analyses, the statistics are expressed as estimated mean differences between typical and atypical groups, with 95% confidence intervals in brackets, *t* values, and *P* values. WMH: White matter hyperintensity.

## Discussion

We empirically identified two distinct cognitive profiles in individuals with MCI due to AD: a typical profile (56.04% of participants) characterized by worse performance on memory than non-memory tests, and an atypical profile (43.96% of participants) with a more uniform pattern of impairment across cognitive domains. These profiles were associated with different clinical trajectories and topographic patterns of atrophy despite similar baseline global cognitive impairment (i.e., MMSE and CDR-SB), age, education, sex distribution, APOE ε4 allele frequency, and baseline levels of biomarkers for AD (i.e., Aβ_42_, Aβ_42/40_ ratio, and p-tau181) and neurodegeneration (i.e., NfL and neurogranin) in CSF. These results show that clinically meaningful typical and atypical cognitive subtypes previously described in mild to moderate AD dementia (Scheltens et al., 2016, 2017; Blanken et al., 2019; Qiu et al., 2019; Habes et al., 2020) are also observed in the early prodromal stage of the disease. Furthermore, results support the position that cognitive subtypes detected in previous studies of clinically defined MCI are characteristic of AD and, as shown in the sensitivity analyses, not caused by the inclusion of individuals with other common non-AD pathologies (e.g., synucleinopathy, white matter lesion pathology, or depression).

The distinct patterns of neuropsychological deficits we observed were associated with different patterns of atrophy observed via neuroimaging. While both MCI subtypes had lower volumes than cognitively unimpaired people in all assessed brain regions, the typical MCI subtype had lower baseline hippocampal and entorhinal cortex volumes than the atypical subtype, reflecting the critical role of those regions in AD pathology (Rao et al., 2022). This finding is consistent with the disproportionate deficits in memory (relative to other cognitive functions) in the typical MCI subtype—these medial temporal lobe structures are critical for memory formation (Squire and Zola-Morgan, 1991) and are the very sites usually involved early in the AD pathological cascade (Dickerson and Sperling, 2008; Wolk et al., 2017; Ossenkoppele et al., 2022; Pontecorvo et al., 2022). In contrast, whole-brain volume or volume of the lateral ventricles and other cortical regions (e.g., middle temporal or fusiform gyrus) did not differ between the two MCI subtypes despite greater impairment on non-memory cognitive tests for the atypical subtype (perhaps because differences in deficits between the two subtypes were less distinct for non-memory measures than for memory measures).

The typical MCI subtype was also associated with greater loss of brain volume over the subsequent four years (i.e., faster atrophy) than the atypical subtype when considering the whole brain, hippocampus, entorhinal cortex, and fusiform cortex. This accelerated atrophy across multiple brain regions suggests a more aggressive neurodegenerative process in the typical MCI subtype. Consistent with this possibility, the risk of progressing to dementia was higher in the typical subtype than in the atypical subtype, and when progression to dementia occurred within the next four years, it was faster. The cognitive subtypes did not, however, differ significantly in rate of decline on the ADAS-Cog13 or MMSE, and there was only a trend for the typical cognitive subtype to worsen faster than the atypical cognitive subtype on the CDR-SB. The lack of significant difference in decline on these relatively coarse screening measures could have resulted from underpowered analyses or an insensitivity of the measures, especially relative to the neuropsychological tests, to cognitive decline in prodromal and mild dementia stages of AD. Studying the rate of cognitive decline in the two subtypes using more rigorous cognitive tests of memory and non-memory processes, independent of the tests used in making the subtype classification, is warranted in the future.

We found no significant differences in CSF levels of Aβ_42_, the Aβ_42_/Aβ_40_ ratio, or p-tau181 levels between the two MCI cognitive subtypes, which suggests they had similar levels of underlying AD pathology. Thus, greater memory impairment and lower baseline hippocampal/entorhinal cortex volumes in the typical MCI subtype implies that brain regions critical for memory function are more vulnerable to the neurodegenerative effects of AD pathology in that subtype than in the atypical subtype. Selective vulnerability of the hippocampus or regions of the neocortex has been described in neuropathological studies of AD and those with “hippocampal sparing” (e.g., a lower ratio of hippocampal to neocortical tangles) are more likely to have an atypical cognitive profile with relatively less salient memory impairment (Murray et al., 2011; Smirnov et al., 2022). Our results are consistent with this finding and suggest that a degree of selective vulnerability of the hippocampus or neocortex may begin in the earliest stages of the disease. However, these results need to be confirmed with a more direct measurement of the distribution of AD pathology in the brains of individuals with prodromal AD using amyloid and tau positron emission tomography (Jack et al., 2020; Aksman et al., 2023; Agostinho et al., 2024).

Previous research demonstrated the benefit of refined, robust clustering methodology for analyzing neuropsychological test data for empirically identifying clinically meaningful subgroups of individuals with MCI (Clark et al., 2013; Bondi et al., 2014; Eppig et al., 2017; Edmonds et al., 2021, 2024). We extend these methods to show that those subtypes occur in MCI with CSF biomarker-confirmed AD. The bootstrap validation approach we employed following model-based clustering of PCA factor scores consistently replicated these distinct cognitive subtypes, providing strong evidence for the stability and reliability of our classification method over the aging MCI-AD continuum. High within-cluster co-occurrence and low between-cluster co-occurrence strongly supports the validity and reproducibility of our two-cluster solution, and suggests that the identified subgroups represent genuine, distinct patterns in the data rather than artifacts of the clustering algorithm or sampling variability. This validation not only reinforces the validity of our findings but also supports the broader applicability of this approach to characterizing MCI heterogeneity.

The ability to identify distinct cognitive profiles that predict disease course in MCI due to AD has significant implications for clinical practice and research. From a clinical perspective, it could facilitate earlier identification of individuals with prodromal AD who are at higher risk of rapid cognitive decline, improving the ability to predict individual disease trajectories and response to interventions. From a clinical research perspective, it could account for cognitive heterogeneity within study groups, and can therefore be used to increase the sensitivity of clinical trials to detecting treatment effects. It could also help guide the selection of more targeted and effective cognitive or neuroimaging outcome measures.

A key strength of this study is its focus on MCI in the context of biomarker-confirmed AD pathology. This focus mitigates potentially confounding effects of including individuals with non-AD etiologies in investigations of how distinct cognitive profiles relate to neurodegenerative processes in the MCI stage of AD. Confirmation of AD via CSF biomarkers does not, however, preclude the possibility that concomitant non-AD pathologies contribute to differences in cognitive profiles. We addressed this issue through sensitivity analyses in a subset of our cohort, showing that the atypical cognitive subtype had a greater volume of WMH than the typical subtype, but controlling for this factor in analyses did not alter our original findings. In addition, there was no difference in CSF alpha-synuclein levels between the two cognitive subtypes, suggesting that the distinct cognitive profiles we observed are not due to greater concomitant Lewy body pathology in one of the subtypes. Still other non-AD pathologies will require examination to determine their influence on the patterns of cognitive heterogeneity (e.g., LATE, PART, hippocampal sclerosis, and cerebral amyloid angiopathy). While the limited availability of non-AD biomarkers in our sample necessitates caution in interpretation, sensitivity analyses with the available non-AD biomarkers we examined reinforce our conclusion that cognitive heterogeneity is a feature of MCI due to AD that can be used to identify clinically meaningful cognitive subtypes that influence disease course.

Although our analyses indicate that the cognitive subtypes are not explained by differences in CSF AD biomarkers, demographic factors, or the comorbid pathologies examined, the precise mechanisms underlying the development of typical versus atypical profiles warrant further exploration. Potential contributors may include genetic modifiers beyond APOE ε4, variations in early-life cognitive reserve, or differential susceptibility of neural networks to AD pathology, as suggested by prior neuropathological evidence of selective vulnerability (Mahon and Lachman, 2022; Gouilly et al., 2023; Moore et al., 2023; Levin et al., 2024). Elucidating these factors could provide insights into the biological basis of cognitive heterogeneity. Furthermore, given the more favorable prognosis associated with the atypical subtype, future studies might investigate whether targeted interventions could modulate cognitive profiles, potentially shifting individuals toward a less aggressive trajectory, though this remains a speculative goal requiring empirical validation.

This study has some limitations that should be noted. First, the participant cohort was predominantly Caucasian and highly educated, which is not representative of the general population from which it was drawn. Generalizability of these findings is therefore limited. Replication of these analyses in more representative cohorts, particularly those with greater diversity of race and ethnicity, socioeconomic and sociocultural background, and educational attainment will be an important next step in affirming the diagnostic and prognostic utility of cognitive heterogeneity in persons with MCI due to AD. Second, our cognitive subtype classification, while internally robust through extensive bootstrap validation, was not externally validated in an independent dataset. Future studies should validate these cognitive subtypes in independent MCI cohorts and develop standardized classification algorithms that can be applied across different research settings and clinical populations. Additionally, our requirement for CSF biomarker confirmation resulted in the exclusion of participants without available CSF data. While our analyses indicated that selection patterns varied across ADNI waves, with some differences offsetting others, we cannot completely rule out selection bias, which may limit generalizability to all MCI populations. Future research should investigate whether similar cognitive heterogeneity exists in broader MCI populations, but rather than requiring invasive biomarker procedures, could assess plasma biomarkers or neuroimaging markers of AD pathology.

In conclusion, our findings have potential applications at multiple stages of clinical assessment and management. At initial MCI diagnosis, cognitive subtyping could enhance prognostic counseling by identifying patients at higher risk for accelerated decline, informing precision medicine approaches (Kivipelto et al., 2020; Arafah et al., 2023). The typical MCI cognitive profile (predominantly memory impairment) appears to identify a group with nearly twice the risk of progression to dementia within 4 years, despite similar baseline severity and biomarker status. This could inform decisions regarding more aggressive monitoring or earlier intervention strategies. For clinical trials, identifying these subtypes at enrollment could allow for more precise stratification, potentially increasing statistical power by reducing heterogeneity in progression rates. Beyond initial diagnosis, this subtyping approach may also prove valuable for monitoring purposes because differential rates of volumetric brain changes suggest distinct underlying pathophysiological processes that might respond differently to emerging treatments. Furthermore, this subtyping can be derived from standard neuropsychological assessments without requiring specialized biomarkers, making it potentially accessible in various clinical settings.

## Additional files:

***Additional Figure 1:*** *Estimated power curve for the Clinical Dementia Rating Sum of Boxes (CDR-SB) linear mixed effects model.*

Additional Figure 1 Estimated power curve for the Clinical Dementia Rating Sum of Boxes (CDR-SB) linear mixed effects model.
The power for a range of sample sizes was estimated by 1000 Monte Carlo repeats for the CDR-SB linear mixed effects model to detect a significant difference of 0.09 in CDR-SB decline rates between typical and atypical groups at α = 0.05.

***Additional Table 1:*** *Institutional Review Boards that provided ethics approval for the study procedures of the Alzheimer’s Disease Neuroimaging Initiative (ADNI) cohorts.*

***Additional Table 2:*** *Comparisons of ADAS-Cog13 subtest scores between the identified MCI subgroups.*

***Additional Table 3:*** *Comparisons between primary analysis and WMH-adjusted sensitivity analysis.*

## Data Availability

*The datasets supporting the conclusions of this article are available in the publicly available ADNI database. The datasets used and/or analyzed during the current study are available from the corresponding author on reasonable request.*
